# Assessing age-related changes in brain activity during isometric upper and lower limb force control tasks

**DOI:** 10.1007/s00429-024-02866-5

**Published:** 2024-12-17

**Authors:** Abigail E. Bower, Jae Woo Chung, Roxana G. Burciu

**Affiliations:** 1 Department of Kinesiology and Applied Physiology, University of Delaware, Newark, DE USA; 2 Department of Neurology, University of Minnesota Medical School, Minneapolis, MN USA

**Keywords:** fMRI, Aging, Force, Upper limb, Lower limb

## Abstract

Despite the widespread use of older adults (OA) as controls in movement disorder studies, the specific effects of aging on the neural control of upper and lower limb movements remain unclear. While functional MRI paradigms focusing on hand movements are widely used to investigate age-related brain changes, research on lower limb movements is limited due to technical challenges in an MRI environment. This study addressed this gap by examining both upper and lower limb movements in healthy young adults (YA) vs. OA. Sixteen YA and 20 OA, matched for sex, dominant side, and cognitive status, performed pinch grip and ankle dorsiflexion tasks, each requiring 15% of their maximum voluntary contraction. While both groups achieved the target force and exhibited similar force variability and accuracy, OA displayed distinct differences in force control dynamics, with a slower rate of force increase in the hand task and a greater rate of force decrease in the foot task. Imaging results revealed that OA exhibited more widespread activation, extending beyond brain regions typically involved in movement execution. In the hand task, OA showed increased activity in premotor and visuo-motor integration regions, as well as in the cerebellar hemispheres. During the foot task, OA engaged the cerebellar hemispheres more than YA. Collectively, results suggest that OA may recruit additional brain regions to manage motor tasks, possibly to achieve similar performance. Future longitudinal studies that track changes over time could help clarify if declines in motor performance lead to corresponding changes in brain activation.

## Introduction

One of the most pressing issues facing the world today is the growing aging population. Advances in medicine and public health over the past few decades have significantly increased average life expectancy and, as a result, a larger percentage of the population is living into their 70-80 s, and beyond (Buxbaum et al. 2020; Crimmins 2015; Dwyer-Lindgren et al. 2022; Woolf and Schoomaker 2019). While increasing the average lifespan is undoubtedly a net positive, elderly individuals face an increased risk of neurodegenerative disorders such as Alzheimer’s disease (Fjell et al. 2014; Henderson 1988; Riedel et al. 2016; Rocca et al. 1986; West et al. 1994) and Parkinson’s disease (PD) (De Lau and Breteler 2006; Dorsey et al. 2018; Feigin et al. 2019; Mayeux et al. 1995; Van Den Eeden 2003). The prevalence of these disorders in the elderly population is substantial, underscoring their significant impact on health and quality of life. Many, if not most, studies examining the effects of neurodegenerative diseases affecting movement, such as PD, rely on age-matched healthy controls to interpret their findings (Burciu et al. 2015; Chung et al. 2023b, 2023a; Herz et al. 2014, 2021; Planetta et al. 2015). However, because there is considerable overlap between pathological symptoms of PD and normal signs of aging (Collier et al. 2017) it is essential to thoroughly understand the effects of aging to distinguish between typical aging processes and disease-related changes.

Much of the impact of aging is felt in the domains of mobility, motor control and coordination, which are critical to maintaining a high quality of life. Previous research has indicated that older adults (OA) experience a decrease in muscle mass and strength (sarcopenia) (Goodpaster et al. 2006; Kim et al. 2018; Pearson et al. 1985; Wilkinson et al. 2018), and speed (Jiménez-Jiménez et al. 2011; Lamb et al. 2016; Salthouse 2000), along with an increase in movement variability (Christou 2011; Darling et al. 1989; Newell et al. 2009; Sosnoff and Newell 2006; Vaillancourt 2003). Research studies have shown that upper limb tasks can often be performed with greater precision and less variability compared to lower limb tasks, suggesting that neurophysiological mechanisms may differ significantly between these domains. For instance, Christou and colleagues (Christou et al. 2003) found that the standard deviation of peak force and impulse was approximately 25% greater for the lower limb than the upper limb, indicating superior force control in the upper limb. This disparity suggests that distinct motor control strategies may govern the movements of the upper and lower limbs, necessitating further investigation into the neural mechanisms involved, particularly as these may be influenced by the aging process.

Thus far, multiple functional neuroimaging studies employing functional magnetic resonance imaging (fMRI) have explored how aging affects neural control of movement, revealing significant alterations in brain activation patterns associated with various tasks. The hemispheric asymmetry reduction in OA model (HAROLD) proposes that with aging, there is a decrease in the typical lateralization of brain activity (Cabeza 2004; Cabeza et al. 1997; Dolcos et al. 2002; Rajah and D’Esposito 2005). In young adults (YA), certain tasks often engage one hemisphere more than the other. However, as individuals age, there is a shift toward more bilateral brain activation, particularly in the prefrontal regions (Cabeza 2004; Dolcos et al. 2002). Initially proposed to explain changes in cognitive functions such as memory, the HAROLD model has since been extended to encompass motor control and other aspects of brain function. Studies comparing motor-related brain activity patterns between YA and OA often reveal that aging is linked to increased activation in a broad range of brain areas, beyond the primary motor cortex (Heuninckx et al. 2005; Mattay et al. 2002; Riecker et al. 2006; Ward 2003). It was noted that this heightened activation tends to extend into regions typically associated with cognitive functions, including the dorsolateral prefrontal and the inferior parietal cortices (Goble et al. 2010). Some studies, including those by Mattay and colleagues (2002) and Ward and Frackowiak (2003), suggest that increased activation in sensorimotor areas ipsilateral to the limb used in the task and the contralateral cerebellum may positively impact task performance through compensatory mechanisms (Mattay et al. 2002; Ward 2003). Of note, the Compensation-Related Utilization of Neural Circuits Hypothesis (CRUNCH) proposes that this increased activation reflects the aging brain’s need for additional neural resources to achieve comparable behavioral outcomes (Reuter-Lorenz and Cappell 2008; Van Ruitenbeek et al. 2023). However, an alternative perspective argues that this increased activation may reflect inefficient neural recruitment rather than compensatory mechanisms. Koen and Rugg (2019) propose that dedifferentiation—reduced efficiency in recruiting the appropriate neural resources—could account for the observed increase in extra-motor activation in OA. Another study by Fujiyama and colleagues offers the idea that this increase in diffuse activity is a result of the loss of inhibitory control in OA (Fujiyama et al. 2009).

Importantly, most studies on this phenomenon have focused on upper limb tasks. Studying lower limb movements in an MRI environment is challenging due to the increased head motion associated with these tasks compared to upper limb movements. This head motion can interfere with data acquisition and undermine the reliability of the findings. For lower limb research, motor imagery has been employed as a method to circumvent the challenges, with some studies showing a similar pattern of activity in OA despite the lack of overt movement (Allali et al. 2014; Mouthon et al. 2018; Wai et al. 2012; Zwergal et al. 2012). Although useful, the primary limitation of motor imagery is that it fails to capture the physical dynamics of actual movements. It does not account for crucial factors such as the force produced, range of motion during tasks like foot tapping, the number of trials performed (e.g., taps), and rhythmicity, which are essential for a comprehensive understanding of motor function in YA vs. OA. Additionally, the effectiveness of motor imagery can be compromised in individuals with cognitive impairments, as their ability to imagine movements may be diminished. While some studies have explored coordination tasks involving simultaneous hand and foot movements (Heuninckx et al. 2005, 2008, 2010; Van Impe et al. 2009), further research that integrates advanced instrumentation with fMRI could offer deeper insights into how motor function evolves with aging. Recently, our group addressed these challenges in a Parkinson’s disease study by using an MRI-compatible ankle dorsiflexion device and force sensors combined with an isometric force control task. This setup allowed for precise force measurement within the scanner while effectively minimizing head motion (Chung et al. 2023b).

Building on these advancements, our study aimed to compare fMRI activity during both upper and lower limb tasks in healthy YA and OA. We hypothesized that, similar to findings in the upper limb, brain activity related to lower limb function in OA, will exhibit a diffuse pattern, extending beyond traditional motor areas involved in movement execution. Specifically, we expect heightened activity in visuomotor regions and areas associated with movement planning and attentional processes, encompassing the frontal, parietal, and cerebellar cortices. Additionally, we anticipated that OA will exhibit a slower rate of force production in both the upper and lower limb tasks and increased variability compared to YA.

## Materials and methods

### Participants

Data from 20 healthy OA (mean age: 63.85 ± 9.46; 10 males, 10 females) and 16 healthy YA (mean age: 26.25 ± 2.84; 7 males, 9 females) was included in this study (see Table 1 for other cohort characteristics). Initial recruitment included 20 YA, but data from 4 YA were excluded due to significant head motion during the lower limb task. The details in Table 1 and throughout the manuscript are based on the final sample of 20 OA and 16 YA. Participants had no history of neurological, neuropsychiatric, or musculoskeletal disorders and were recruited from the local community in Newark, Delaware. Sixteen of the OA participants had previously served as control participants in a study investigating how PD affects fMRI activity associated with force control during a multi-limb coordination task (Chung et al. 2023a) All participants were screened for cognitive impairment with the Montreal Cognitive Assessment (MoCA; Nasreddine et al. 2005), and for depression with the Beck Depression Inventory (BDI) (Beck et al. 2011). Importantly, both groups demonstrated normal cognitive function and no signs of depression, factors that could otherwise affect motor performance. Additionally, OA underwent further screening with part III of the Movement Disorder Society Unified Parkinson’s Disease Rating Scale (MDS-UPDRS) to exclude the presence of motor symptoms associated with PD, which is common in OA (Goetz et al. 2008). The OA and YA groups were comparable in terms of sex distribution, handedness, tested side, and scores on MoCA and BDI, as detailed in Table 1. All participants provided written informed consent, and the study procedures were approved by the local Institutional Review Board, adhering to the 1964 Helsinki Declaration.

**Table 1:** Summarizes the characteristics of the YA and OA cohorts

Cohort Characteristics	YA	OA	P-Value
N	16	20	n/a
Age (Years)	26.25 (2.84)	63.85 (9.46)	< 0.001*
Sex (Male | Female)	7 | 9	10 | 10	0.709
Tested Side (Left | Right)	1 | 15	3 | 17	0.406
Tested Side (Dominant | Non-Dominant)	8 | 8	13 | 7	0.364
Education Level (Years) †	17.13 (1.20)	16.65 (2.91)	0.404
Montreal Cognitive Assessment Test †	28.81 (1.42)	26.95 (2.91)	0.053
Beck Depression Inventory – II †	5.50 (8.03)	5.05 (6.37)	0.838
MVC Tested Hand (N)	77.56 (23.25)	61.10 (24.48)	0.048*
MVC Tested Foot (N)	106.69 (58.53)	78.90 (45.96)	0.120

Values are presented as either counts or means (± SD). The last column shows *p*-values, with significance set at *p* < 0.05. *MVC* Maximum voluntary contraction, *N* Number (or Newton in the case of MVC), *OA* Older adults, *YA* Young adults

A dagger (†) indicates variables for which non-parametric statistics were used

*Significant at p < 0.050; † indicates non-parametric statistics

### Force data collection

**Fig. 1:**
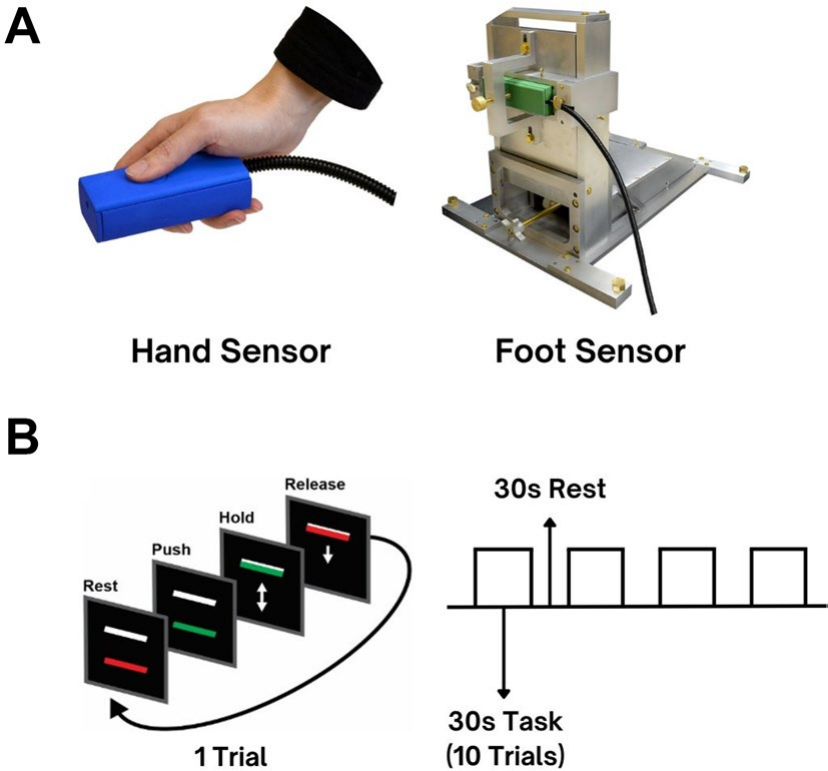
Contains: **A** The MRI-compatible hand sensor (left) and foot device along with foot sensor (right) that were utilized in the force data collection. **B** A visual representation of the force paradigm. Participants were tested on each limb separately, as detailed in the methods (the task order was randomized). They were instructed to exert 15% of their maximum voluntary contraction (MVC) for 2 s, followed by 1 s of rest between trials, for both hand and foot tasks. Each fMRI session began with 30 s of rest, followed by four cycles of 30 s of force followed by 30 s of rest. Each force block consisted of 10 trials (totaling 30 s). The task was visually guided, with a white bar indicating the force target (set at 15% of MVC) and a colored bar representing the force being produced. Participants were instructed to relax when the bar was red and to generate force when the bar was green

The data collection methods and isometric force paradigms—specifically pinch grip and ankle dorsiflexion – have been effectively employed in our previous research on PD, as well as in studies conducted by others in the field. Such paradigms successfully engage the brain networks involved in upper limb movements (Burciu et al. 2015, 2016; Chung et al. 2023a; Prodoehl et al. 2010; Spraker et al. 2010) and lower limb movements (Chung et al. 2023b, 2023a; See et al. 2021). The pinch grip task specifically engages the neural circuits involved in fine motor control, which are essential for daily activities such as grasping and manipulating objects. Similarly, the ankle dorsiflexion task described below is integral to the gait cycle and is essential for maintaining balance. Importantly, the consistent application of pinch grip and ankle dorsiflexion tasks across various research investigations helps enhance the validity of findings and facilitates meaningful comparisons with existing literature. In the current study, force data were collected with participants lying horizontally on the MRI scanner bed. Hand data were collected using an MRI-compatible fiber-optic force transducer held in the tested hand, with participants applying isometric force against an indented button (Neuroimaging Solutions, Gainesville, FL; Fig. 1A). Foot data were collected using a similar fiber-optic force transducer that was inserted into a custom MRI-compatible device into which the tested foot was placed (Fig. 1A). The device was designed to minimize head motion by using an adjustable strap placed over the metatarsals. This setup allowed for isometric dorsiflexion of the ankle. With each ankle dorsiflexion, a piston on the back of the foot device applied force to a fiber-optic force transducer. Force output was displayed to participants through real-time feedback projected onto a 32″ 1920 × 1080 widescreen LCD with a 120 Hz refresh rate. The screen was positioned behind the participant’s head outside the MRI bore and was visible using a mirror attached to the MRI head coil. Force signals and output were transmitted via a fiber-optic cable to an SI155 Micron Hyperion Optical Sensing Interrogator (Micron Optics, Atlanta, GA) in the MRI control room, where the force data were digitized. Data collection was performed using custom software designed in LabVIEW (National Instruments, Austin, TX).

### fMRI force protocol

All participants received training on the paradigm outside the MRI before beginning the study. Maximum voluntary contraction (MVC) was measured for both the hand and foot independently. A single bar was projected onto the computer screen, and participants were instructed to press with their thumb or dorsiflex their ankle with maximum effort when the bar turned green, and to relax when it turned red. This process was repeated three times, with each trial lasting 5 s and breaks allowed between trials. The MVC was used to determine the force required for the paradigm, which was set at 15% of the MVC. This normalization approach, based on previous studies involving OA (Chung et al. 2023b, 2023a), ensures consistent force production demands across participants and helps to minimize excessive head motion, as the foot generally generates more force than the hand. Inside the MRI scanner, participants were instructed to view a black screen on which two bars were displayed (Fig. 1B). The top white bar was stationary and represented the target force level (15% of MVC), while the bottom bar was movable and displayed the force produced by the participant. When the bar turned green, participants were instructed to press down with their thumb or dorsiflex their ankle, depending on whether they were completing the hand or foot task, to match the target bar as quickly and accurately as possible, and relax when the bar returned to red. Participants received visual feedback on their task performance throughout the experiment. The fMRI design was a block design that began with 30 s of rest, followed by four cycles of 30 s of force production and 30 s of rest. Each force block consisted of 10 trials, with each force production lasting for 2 s followed by a 1-s rest period between trials (Fig. 1B). This rapid contraction and relaxation sequence was designed to actively engage motor regions at both cortical and subcortical levels (Burciu et al. 2015, 2016; Chung et al. 2023a, 2023a; Prodoehl et al. 2010; Spraker et al. 2010). The alternating periods of force production and rest stimulate neural activity in the primary motor cortex and premotor cortices, as well as subcortical structures involved in motor control, such as the basal ganglia and cerebellum. Participants were tested on either the left or right side, as detailed in Table 1. The sequence of tasks was randomized to control for order effects.

### Force data analysis

Force data were checked in real-time during the paradigm to ensure participants were able to follow directions and produce force for all 40 trials in each task. Post-acquisition analyses were done using custom scripts in MATLAB R2021b (The Mathworks, Natick, MA), following procedures consistent with those employed in previous force studies (Chung et al. [Bibr CR13][Bibr CR15], 2023a; Neely et al. 2013). After data were filtered using a 6th-order Butterworth filter with a cutoff frequency of 15 Hz, the following four time points were determined for each trial: 1) initiation of force, 2) onset of the steady period, 3) offset of the steady period, and 4) cessation of force. These time points were used to evaluate three critical aspects of force production. Force amplitude was measured as the peak force exerted, expressed as % MVC. Force production speed was assessed by calculating the rate of force increase and decrease, with both measures normalized to MVC (i.e., % MVC/s). Finally, force precision was assessed by calculating the absolute error in Newtons and the standard deviation (SD) of force, normalized to MVC, to evaluate how accurately participants met the target.

### MRI data collection

MRI images were collected on a Siemens 3 T Magnetom Prisma whole-body MRI scanner with a 64-channel head coil at the University of Delaware’s Center for Biomedical and Brain Imaging in Newark, DE. To minimize head motion during the scans, padding was used around the head to stabilize and support it. Participants were instructed not to move their head or body throughout the procedure. A small pillow was placed under the knee to ensure a more comfortable and stable position during the ankle task. High-resolution T1-weighted structural images were collected using the following parameters: TR = 2000 ms, TE = 2.99 ms, 208 slices, flip angle = 8°, GRAPPA parallel imaging factor 2, FOV = 256 × 256 mm, and resolution = 0.8 × 0.8 × 0.8 mm. Functional MRI data were acquired with a single-shot gradient echo-planar imaging sequence: TR = 2500 ms, TE = 30 ms, 43 slices, flip angle = 80°, GRAPPA parallel imaging factor 2, FOV = 240 × 240 mm, and resolution = 3 × 3 × 3 mm. Finally, T2-weighted structural scans were collected to rule out any structural abnormalities that may be less apparent in T1-weighted scans. These scans were acquired with the following parameters: TR = 2500 ms, TE = 371 ms, 208 slices, flip angle = variable, GRAPPA parallel imaging factor 3, FOV = 256 × 256 mm, and resolution = 0.8 × 0.8 × 0.8 mm.

### fMRI data analysis

The fMRI data were analyzed using several software packages, including AFNI (Analysis of Functional Neuroimages; https://afni.nimh.nih.gov), SPM12 (Statistical Parametric Mapping; https://fil.ion.ucl.ac.uk/spm/), and custom UNIX shell scripts. All MRI images were thoroughly evaluated for quality prior to any analysis. Consistent with prior research on unilateral movements (Burciu et al. 2015, 2016; Chung et al. 2018, 2023b; Planetta et al. 2015) functional and T1-weighted scans of participants tested on their left side were flipped along the X axis before processing. From this point forward in the results and discussion, references to the left side of the brain will denote the hemisphere contralateral to the tested limb, while references to the right side will denote the hemisphere ipsilateral to the tested limb. First, the T1-weighted scan was skull-stripped. The preprocessing for fMRI scans included removing extreme time series outliers through despiking, correcting for slice-time acquisition, and applying 3D rigid-body motion correction. Motion scrubbing was performed to eliminate TR-to-TR motion exceeding 0.5 mm. The fMRI scan was then coregistered to the T1-weighted scan. Signal normalization involved dividing the fMRI signal of each voxel at each time point by the mean signal of that voxel across the entire scan. Spatial normalization was performed to align individual scans with standardized brain templates. For whole-brain analysis, we utilized the 152 MNI template, which is a commonly used reference for standardizing brain images across different subjects. For cerebellar and brainstem analyses, we employed the SUIT (Spatially Unbiased Infratentorial Template; https://www.diedrichsenlab.org/imaging/suit.htm), specifically designed to improve the alignment of the infratentorial regions of the brain. This targeted approach minimizes inter-subject anatomical variability, increasing sensitivity at detecting robust cerebellar activity. Finally, the normalized scans were smoothed with a 4 mm Full Width Half Maximum (FWHM) Gaussian kernel to reduce intersubject variability in neural anatomy. As noted earlier in the methods section, the study was initially designed to include 20 participants from each group. However, 4 young participants had to be excluded due to excessive head motion during the foot task, which was consistent throughout the active/force blocks rather than being isolated outliers. This resulted in a final sample size of 16 YA. The challenges associated with measuring lower limb force, even at relatively low force levels, are discussed in detail in the results and discussion sections, clarifying the difficulties and implications for future research in this area of MRI measurement. Once preprocessing of the scans was completed, the fMRI signal was modeled using a boxcar regressor convolved with the canonical hemodynamic response function. Head motion parameters from the 3D rigid-body motion correction were included as regressors of no interest in the model. Voxel-wise Independent T-Tests were conducted to compare hand- and foot-related fMRI activity between OA and YA. Type I error was controlled using AFNI’s 3dClustSim, which provided a p-value and minimum cluster size threshold equivalent to a family-wise error (FWE) *p* < 0.05 correction. Briefly, we utilized 3dFWHMx to calculate the autocorrelation function (ACF) parameters from the subject-level residuals using whole-brain mask for the supratentorial analysis and the SUIT mask for the infratentorial analysis. These averaged parameters across subjects were then used in the 3dClustSim simulation which established a minimum cluster size of 7 voxels (or 189 µL) for the whole-brain analysis and 4 voxels (or 108 µL) for the cerebellar/SUIT analysis, with a statistical threshold of *p* < 0.001, applying the 3 nearest neighbors (NN; face + edge + corner) method. Regions that met the threshold for significance were labeled using the basal ganglia human area template (BGHAT), the human motor area template (HMAT), the probabilistic atlas of the human cerebellum (SUIT), and the automated anatomical labeling atlas version 3 (AAL3) (Diedrichsen 2006; Mayka et al. 2006; Prodoehl et al. 2008; Rolls et al. 2020).

### Statistical analyses

Behavioral measures were analyzed using SPSS 29.0 (IBM, New York). All data were tested for normality with the Shapiro–Wilk test and for homogeneity of variance with Levene’s test to ensure the appropriate statistical methods were used. The results of these tests guided the choice between parametric and non-parametric statistical methods. Categorical data, such as sex, handedness, and tested side, were compared using Chi-square tests. Continuous data, such as education level, MoCA scores, BDI scores, and MVC for both hand and foot, were analyzed using Independent T-Tests or Mann–Whitney U Tests, depending on the outcome of the normality tests. Repeated measures analyses were conducted for the force measures, with group as the between-subject factor and limb (hand and foot) as the within-subject factor. Significant group effects, limb effects, and group x limb interactions were further analyzed using either an Independent T-Test for group comparisons or a Paired T-Test for limb comparisons. Results were considered significant if *p* < 0.05.

## Results

### Cohort characteristics

The results, including *p*-values for the statistical analyses, are summarized in Table 1. As expected in an aging study, the two groups differed in age (mean age YA = 26.25 ± 2.84; mean age OA = 63.85 ± 9.46; *p* < 0.001). However, they were well-matched in terms of sex distribution, tested side (whether the tested limb was the dominant or non-dominant side), handedness, education level, and cognitive status, and showed no differences in BDI scores, indicating that both groups were free of depression (*p-*values > 0.05; Table 1).

### Force data

**Fig. 2:**
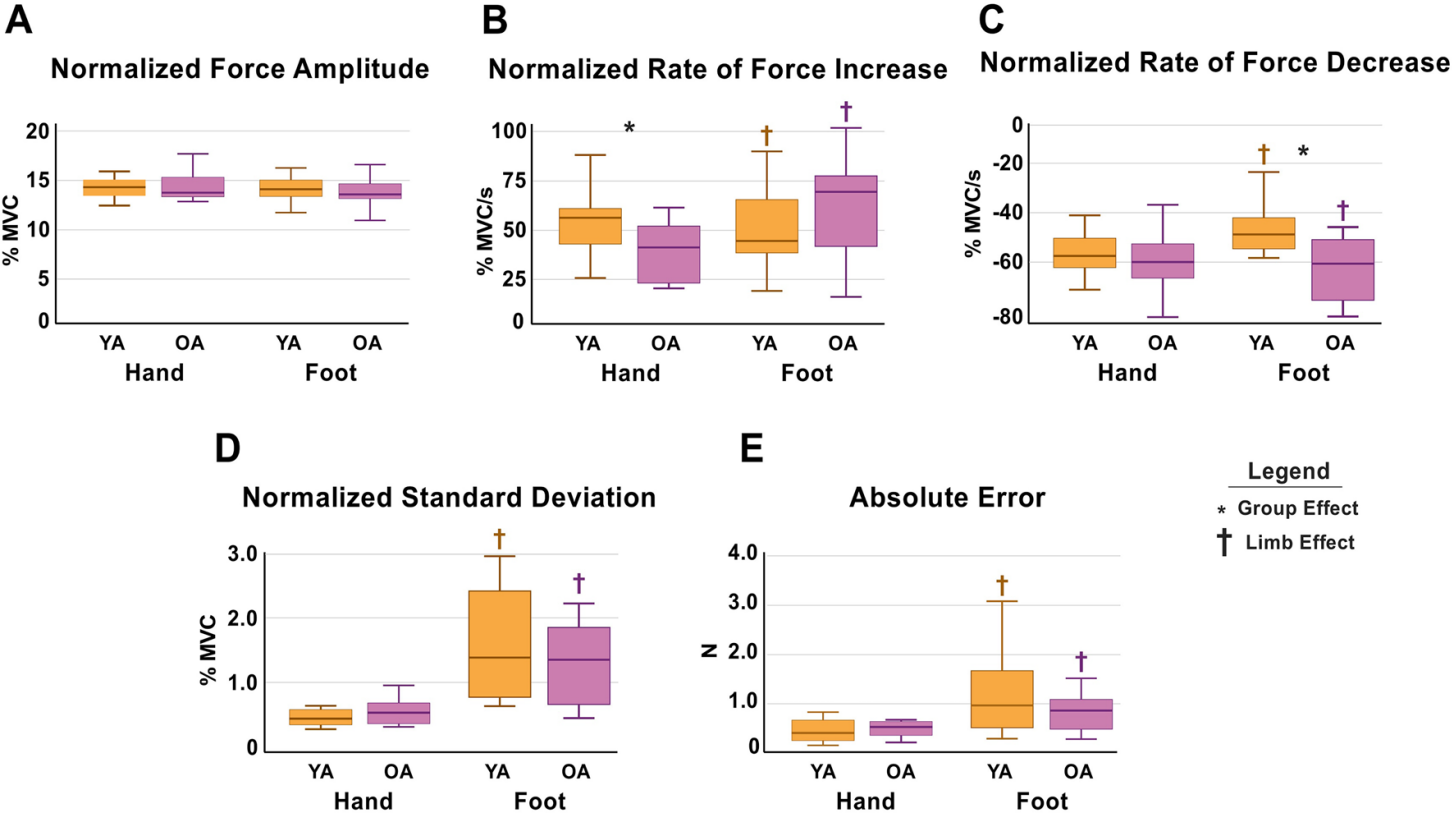
Figure presents the results of the force analysis. An asterisk (*) indicates a significant group effect, while a dagger symbol (†) denotes a limb effect. **A** Normalized mean force amplitude during the hand and foot tasks, reported as a % MVC. **B** Normalized rate of force increase in the hand and foot tasks, expressed as % MVC/s. **C** Normalized rate of force decrease in the hand and foot tasks. **D** Normalized variance (standard deviation) in the hand and foot tasks. **E** Absolute error in the hand and foot tasks. *OA* Old adults, *YA* Young adults

Mean values for each force variable, organized by group and task, are detailed in Table 2. Figure 2 visually represents these results using boxplots, which display the median along with the 25th and 75th percentiles. There were significant group differences in the hand MVC, with OA having slightly lower values compared to YA, while no statistically significant differences were found in the foot MVC (Table 1).

**Table 2:** Presents the results of the force analysis, expressed as mean (± SD)

Force Variables	H–YA	H–OA	F–YA	F–OA
Normalized Force Amplitude (% MVC)	15.02 (0.48)	14.90 (1.23)	14.76 (0.82)	15.35 (2.76)
Normalized Rate of Force Increase (% MVC/s)	56.97 (16.09)	42.18 (15.39)	44.93 (15.49)	58.98 (26.42)
Normalized Rate of Force Decrease (% MVC/s)	− 53.22 (8.81)	− 58.13 (12.99)	− 46.30 (8.25)	− 67.50 (18.26)
SD of Normalized Force Amplitude (% MVC)	0.61 (0.16)	0.68 (0.23)	1.71 (0.74)	1.28 (0.54)
Absolute Error (N)	0.43 (0.23)	0.64 (0.71)	1.25 (0.95)	0.98 (0.58)

*MVC* Maximum voluntary contraction, *N* Newtons, *OA* Older adults, *SD* Standard deviation, *YA* Young adults

The 2 × 2 repeated measures ANOVA revealed no significant differences between YA and OA in normalized force for either hand or foot, as well as no significant limb effect or group x limb interaction (group effect *p* = 0.587; limb effect *p* = 0.779; group x limb interaction *p* = 0.325). That is, both groups were able to produce the target force in each task, which is desirable for an fMRI experiment (Table 2, Fig. 2A).

There were no significant group (*p* = 0.945) or limb effects (*p* = 0.516) for the rate of force increase normalized to MVC. However, a significant group x limb interaction was found (F_[1,34]_ = 15.893, *p* < 0.001). Post hoc tests revealed that OA had a significantly reduced rate of force increase during the hand task compared to YA (*p* = 0.008), but not during the foot task (*p* = 0.069) (Table 2, Fig. 2B). Moreover, YA exhibited a slower rate of force increase during the foot task compared to the hand task (p = 0.011). In contrast, OA displayed the opposite pattern, with a slower rate of force increase during the hand task than the foot task (p = 0.001).

The statistical analysis also revealed the following results for the rate of force decrease normalized to MVC: a significant group effect (F_[1,34]_ = 13.271, *p* < 0.001), a significant group x limb interaction (F_[1,34]_ = 10.126, *p* = 0.003), and no limb effect (*p* = 0.636). Post hoc tests showed that OA were faster at relaxing force during the foot task compared to YA (*p* < 0.001), but no significant group differences were observed during the hand task (*p* = 0.205) (Table 2, Fig. 2C). YA demonstrated a greater rate of force decrease for the hand compared to the foot (*p* = 0.037), while OA showed the opposite pattern with a greater rate of force decrease for the foot than the hand (*p* = 0.010).

With regard to the normalized standard deviation (SD) of force, there was a significant limb effect (F_[1,34]_ = 60.556, *p* < 0.001) and a significant group x limb interaction (F_[1,34]_ = 5.263, *p* = 0.028), but no significant group effect (*p* = 0.124). That is, normalized SD was similar in both groups for the hand (*p* = 0.305) and foot tasks (*p* = 0.052) (Table 2, Fig. 2D). As expected, both YA and OA showed greater normalized SD in the foot task compared to the hand task (YA: *p* < 0.001; OA: *p* < 0.001).

Finally, regarding force accuracy, absolute error revealed significant limb effects (F[1,34] = 26.178, *p* < 0.001) and a significant group-by-limb interaction (F[1,34] = 4.588, *p* = 0.039), with no significant group effect (*p* = 0.893). Post hoc analysis revealed no significant group differences in absolute error for either the hand (*p* = 0.248) or foot tasks (*p* = 0.333). However, in line with the normalized SD results, absolute error was greater during the foot task than the hand task for both YA (*p* = 0.005) and OA (*p* = 0.002).

### fMRI data

**Fig. 3:**
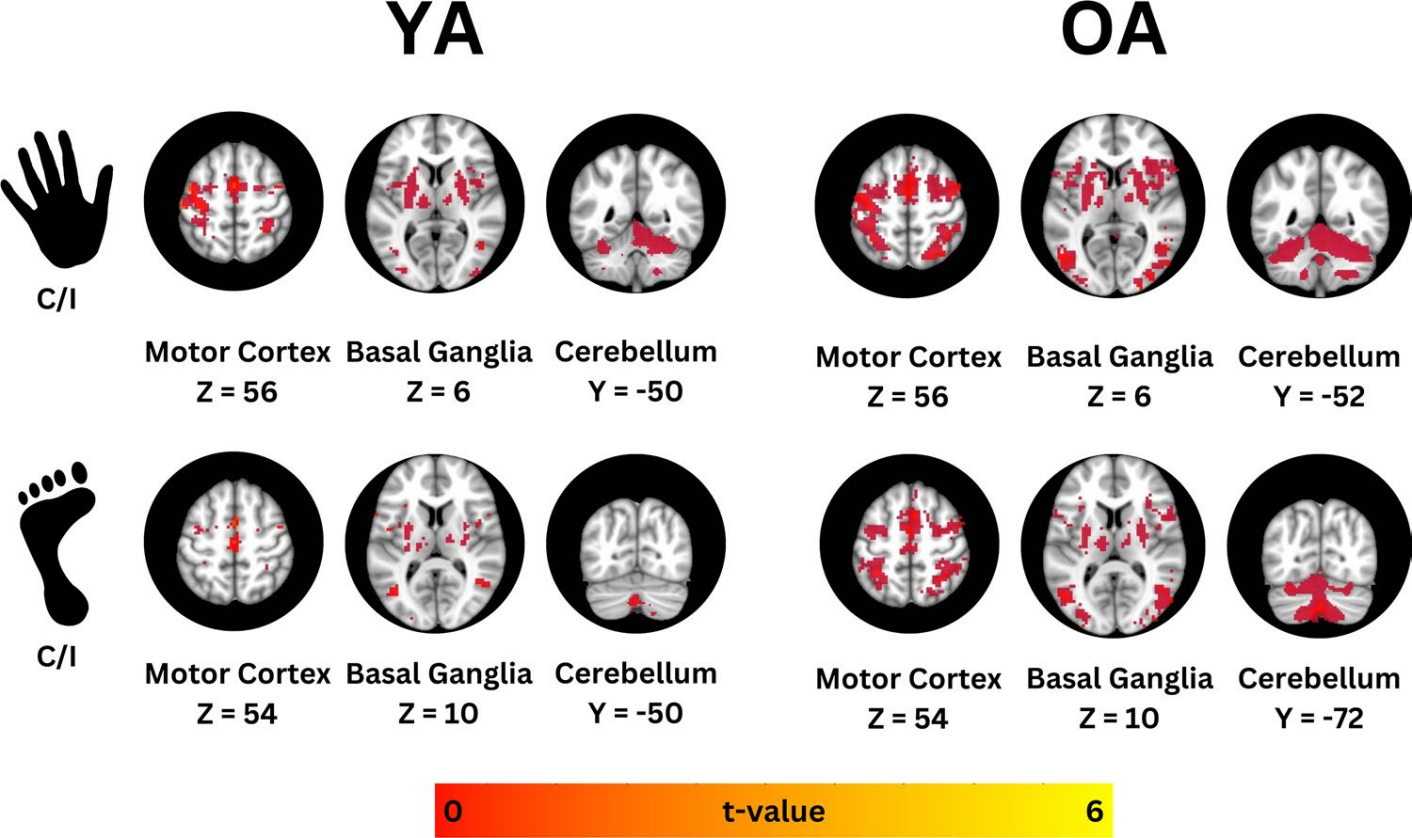
Mean fMRI activity for YA and OA groups during the two force tasks. The top section shows mean fMRI activity during the hand task, while the bottom section shows mean fMRI activity during the foot task. Slices were selected to highlight the extent of activation across key nodes of the motor circuit, including cortical motor regions, basal ganglia, and cerebellum. Results are overlaid on the MNI 152 T1-weighted template, with coordinates provided in MNI space. The color bar represents t-values.* C* Contralateral to the side producing force, *I* Ipsilateral to the side producing force, *OA* Old adults, *YA* Young adults

**Fig. 4:**
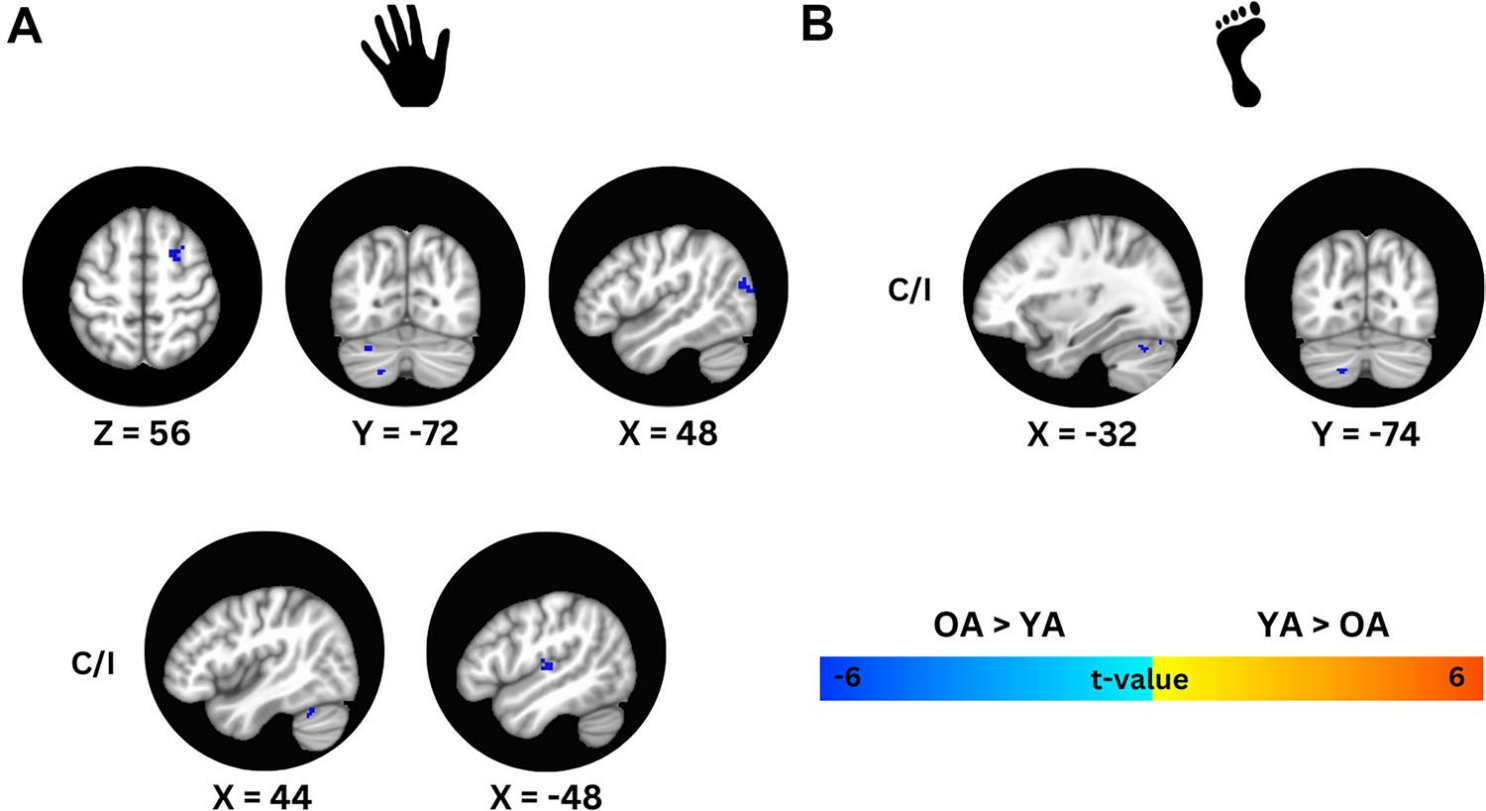
The top section (**A**) shows the results of the voxel-wise independent t-test for the hand task, while the bottom section (**B**) presents the results for the foot task. Results are overlaid on the MNI 152 T1-weighted template, with coordinates provided in MNI space. Cool colors indicate regions where fMRI activity was significantly greater in OA compared to YA. No regions are depicted in warm colors, which would have indicated where fMRI activity was greater in YA. The color bar represents t-values. *C* Contralateral to the side producing force, *I* Ipsilateral to the side producing force, *OA* Old adults, *YA* Young adults

First off, in the analysis of fMRI data, a total of 0.31 ± 0.87 TRs (or ~ 0.28% of the total volumes) were removed for the hand task in the YA group, while 1.15 ± 2.45 TRs (or ~ 1.06% of the total volumes) were removed for the hand task in the OA group. For the foot task, 6.19 ± 11.21 TRs (or ~ 5.73%% of the total volumes) were removed for YA, compared to 2.70 ± 3.24 TRs (or ~ 2.50% of the total volumes) for OA. In Fig. 3, one can observe the mean fMRI activity for each task and group. Across cortical motor and premotor areas, as well as subcortical structures, OA tend to exhibit more widespread activation compared to the YA group, which displays a more focal activity pattern. This pattern is consistent for both hand and foot tasks. A voxel-wise Independent T-Test revealed that during the hand task, OA exhibited greater fMRI activity compared to YA in several regions, including the dorsal premotor cortex (PMd), middle occipital gyrus, middle temporal gyrus, and various areas of the cerebellum, specifically Crus I, Crus II, and lobule VIIb. Increased activity was also observed in the brainstem, particularly in the pons (Fig. 4A). More details about the side, cluster size, and t-values for these regions are available in Table 3. There were no regions where activity was greater in YA compared to OA.

**Table 3:** Presents the results of independent t-tests comparing hand- and foot-related fMRI activity between OA and YA

Brain Regions	Side	Cluster Size	Peak MNI Coordinates (X Y Z)			T-Value
HAND TASK: OA > YA						
PMd	I	26	24	6	57	− 4.71
Middle occipital gyrus, middle temporal gyrus	I	21	51	− 78	18	− 3.87
Cerebellum: Crus I	C	18	− 28	− 74	− 27	− 3.88
Brainstem: Pons	C	14	− 2	− 40	− 39	− 4.17
Cerebellum: VIIb, Crus II	C	13	− 18	− 72	− 45	− 4.11
Cerebellum: Crus I/II	C	9	− 46	− 44	− 41	− 3.77
Middle occipital gyrus	I	8	39	− 81	36	− 4.37
Cerebellum: Crus I	I	7	44	− 54	− 27	− 3.61
FOOT TASK: OA > YA						
Cerebellum: Crus II, VIIb	C	14	− 18	− 74	− 43	− 4.38
Cerebellum: VI, Crus I	C	14	− 32	− 62	− 25	− 3.84
Cerebellum: Crus I	C	6	− 30	− 76	− 23	− 3.76

Type I error was controlled using AFNI’s 3dClustSim, with a statistical threshold of *p* < 0.001 and minimum cluster sizes of 7 voxels (189 µL) for whole-brain analysis and 4 voxels (108 µL) for cerebellar analysis. These parameters ensured a family-wise error (FWE) correction of *p* < 0.05. The results are detailed by brain region, cluster size, and MNI coordinates of the voxel with the highest t-value within each cluster. Notably, for both tasks, fMRI activity was significantly greater in OA compared to YA, with no regions showing greater activity in YA

*C* contralateral to side producing force, *I* ipsilateral to side producing force, *MNI* Montreal Neurological Institute, *PMd* dorsal premotor cortex

In the foot task, OA showed increased fMRI activity primarily within the cerebellum, with notable activation in Crus I, Crus II, and lobules VI and VIIb (Fig. 4B, Table 3). As with the hand task, no regions showed greater activity in YA compared to OA. Importantly, group differences do not represent variations in activation intensity within shared brain regions. Rather, the findings indicate that the regions exhibiting significantly greater activation in OA do not overlap with those activated in YA. Thus, the activations observed in OA are unique to this group rather than simply stronger responses in common areas.

Finally, for all clusters listed in Table 3, we conducted partial correlations to examine the relationship between percent change in the fMRI signal and the variables differing between the two groups—specifically, the rate of force increase during the hand task and the rate of force decrease during the foot task—while controlling for age. Results revealed no significant correlations between the rate of force production or relaxation in any of the brain regions, with all *p*-values exceeding 0.05.

## Discussion

This study aimed to investigate differences in fMRI activity associated with isometric force control during hand and ankle tasks between YA and OA. The study yielded several key findings. As anticipated, both groups produced a similar amount of force for the hand and foot conditions. Although force variability and accuracy were comparable between groups, both YA and OA exhibited greater variability and reduced accuracy during the foot task compared to the hand task. Notable group differences emerged in speed-related measures, with OA having a reduced rate of force increase compared to YA during the hand task, and a greater rate of force decrease during the foot task. Imaging results revealed a more extensive and diffuse pattern of neural activity in OA during both the hand and foot tasks, with greater activation in OA compared to YA extending beyond the brain regions involved in the execution of movement.

It has been well-established that muscle strength decreases with age (Goodpaster et al. 2006; Pearson et al. 1985; Volpi et al. 2004), which is reflected here in the lower hand MVC observed in OA compared to YA. There was also a trend for lower foot MVC in OA, as expected, but this difference did not reach statistical significance, likely due to variability between subjects. Despite the overall decrease in hand strength, OA were able to perform the force production task at 15% of their MVC with similar precision to YA. This was evidenced by similar levels of force variability and accuracy between the two groups.

The observed similarity in force variability and accuracy during the foot task indicates that this cohort of OA maintained intact precision in force control across multiple limbs. While previous research frequently reports increased force variability and decreased force accuracy in OA (Christou 2011; Newell et al. 2009; Sosnoff and Newell 2006; Vaillancourt 2003), our study did not identify such differences, possibly due to specific cohort characteristics or task conditions. Different results might emerge in OA exhibiting subtle cognitive or motor deficits or under varying force demands. Additionally, the nature of activities participants engage in can significantly influence precision in force control. Although not addressed in the current study, collecting detailed information on participants’ exercise habits and activity levels could offer valuable insights into cohort characteristics and their potential impact on force-related measures. Future research should integrate these variables to deepen the understanding of their impact on force performance. Additionally, studies should include older adults with cognitive impairments to evaluate how these conditions influence the distribution of neural activity during motor tasks. Such investigations would offer valuable insights into the relationship between cognitive function and motor control in aging populations.

As anticipated, both groups exhibited greater force variability and reduced force accuracy in the foot task compared to the hand task. This effect is likely influenced by the distinct functional roles of the two limbs: the lower limb is primarily engaged in gross motor tasks, such as walking, whereas the upper limb is often involved in fine motor control (Catani 2017; Christou et al. 2003; Duchateau and Enoka 2022). When interpreting the results, one needs to keep in mind that the MVC was maintained at 15% to facilitate comparison with other studies, minimize head motion during the MRI scans, and enable a direct comparison of force control between limbs. Here, the average MVC in both YA and OA for the foot tended to be greater than the MVC for the hand, and previous work, such as that by Sherwood and Schmidt (1980), has demonstrated that variability tends to increase as the amount of force increases (Sherwood and Schmidt 1980). The observed differences in the rate of force increase in the hand task suggest that while OA can still reach the target force, they do so more slowly, indicating a reduced speed in muscle activation. Interestingly, although not statistically significant, YA tended to be slower than OA in the foot task. Moreover, younger participants were significantly slower in relaxing during this task (Fig. 2). Given that the raw foot MVC was slightly higher in YA compared to OA, YA may have needed to slow down slightly to reach the target force at 15% MVC, despite the emphasis on both speed and accuracy in the task instructions. This adjustment could have been necessary to maintain precision. Similarly, despite producing the same target force of 15% MVC, YA, with a higher raw MVC, might need a more gradual relaxation phase.

Imaging results as highlighted in Fig. 1, revealed that YA exhibited more focal activation across important key nodes of the motor circuit, while OA demonstrated more widespread activation across these same areas, and beyond. While previous studies have shown that YA exhibit not only more localized activation but also greater activation in the contralateral motor regions compared to OA (Hutchinson 2002), our findings did not reveal significant group differences in cortical primary motor or somatosensory cortices activation. This absence of significant differences may stem from the specific characteristics of our cohort. OA from this study served as controls in previous PD research studies where they underwent comprehensive screening for motor dysfunction. Specifically, the motor section of the MDS-UPDRS (i.e., MDS-UPDRS-III) was employed to exclude any signs of parkinsonism, thereby ensuring that participants did not present motor deficits that could potentially confound the results. While administering such clinical scales is not a common practice in aging studies, it could prove beneficial given that aging increases the risk of neurodegenerative diseases like PD (Collier et al. 2011; Hou et al. 2019; Reeve et al. 2014) which can adversely affect motor function. Incorporating these assessments in future research may help mitigate variability in findings related to motor performance and fMRI activity among aging studies, thereby facilitating a more nuanced understanding of age-related changes and their impact on motor control. By adopting a standardized approach to screening, researchers could better account for undetected motor impairments, ultimately enhancing the validity and reliability of conclusions drawn in studies involving older adults.

Several studies have corroborated that aging is associated with decreased activation in the contralateral primary motor and somatosensory cortices (Calautti et al. 2001; Mattay et al. 2002; Naccarato et al. 2006) and increased activation in ipsilateral motor areas (Hutchinson 2002; Riecker et al. 2006; Turesky et al. 2016). Our findings are somewhat consistent with this literature, as we observed increased activation in the ipsilateral dorsal premotor cortex (PMd) among OA. The imaging analysis also revealed that OA exhibited increased activation in regions involved in processing visuomotor information, such as the occipital and temporal cortices. These areas are essential for executing visually guided tasks. The increased activation of visual and premotor areas not only reflects the demands of the task but also supports the HAROLD theory (Cabeza 2004; Dolcos et al. 2002), which posits that aging is associated with reduced lateralization of brain activity and broader activation across regions. Here, the more extensive activation patterns observed in OA (seen during both tasks) confirm previous findings which have associated the increased and widespread activity in OA compared to YA to possible compensatory mechanisms in response to age-related declines in motor function. However, both in this study and previous research, the cross-sectional design highlights a critical limitation: it cannot adequately capture the dynamic changes in motor function and associated brain activity over time. Longitudinal studies are essential to understanding how these variables interact and evolve, providing insights into the trajectory of motor control and changes in brain function in the aging population. Therefore, while the observed activation patterns may suggest compensatory mechanisms, their interpretation should be approached with caution, as without longitudinal data, it is challenging to draw definitive conclusions about the nature and implications of these adaptations. Using partial correlations while controlling for age, we found no significant relationships between force production or relaxation rates and signal intensity in any brain regions where older OA had greater activity than YA. This lack of correlation spurs the development of theories that require testing through more specific study designs. For example, age-related declines in motor performance may be linked to changes in neural activation patterns and the physical capacity to generate force. This could mean that OA might for instance show lower rates of force increases, which could be reflected in both their behavioral output during this experiment and brain activity levels, without a direct compensatory connection from one to another. Furthermore, as individuals age, they may rely on different neural strategies to execute motor tasks, activation patterns that do not correlate with the actual performance metrics. For example, OA might exhibit heightened activation in certain brain regions to maintain task performance, even if they cannot achieve the same rates of force increase as younger individuals. Collectively, these findings highlight the complexity of motor control and brain function in older adults and highlight the need for further research.

Finally, OA exhibited greater activation in the cerebellar hemispheres during the hand and foot tasks compared to YA. This increased activation spanned non-motor areas of the cerebellum, including lobules VI, Crus I-II, and VIIb, which are important for the more cognitive aspects of movement (Roostaei et al. 2014; Schmahmann 2019; Stoodley and Schmahmann 2018). These patterns mirror the cortical activation observed in OA, where areas involved in movement preparation, rather than execution, show increased recruitment. Given the well-established role of the cerebellum in motor control and planning, its activation in both tasks for YA and OA is expected. Linortner and colleagues found a significant correlation between age and cerebellar activation, interpreting this relationship as evidence of age-related compensatory mechanisms. (Linortner et al. 2014). Interestingly, during the foot task, OA showed increased activation primarily in the cerebellum, with no significant cortical differences compared to YA. This suggests that while the role of the cerebellum in motor control and coordination remains critical for both tasks, the foot task might rely less on the cortical areas. The hand task, which requires precise fine motor control, seems to extensively engage both cortical and cerebellar regions. In contrast, while the foot task also requires producing the same force level, it may be less suited to fine control compared to hand movements. This decreased naturalness of the task could lead to reduced reliance on cortical regions and less engagement in detailed planning.

While this study provides valuable insights into changes in the neural control of upper and lower limb movements with aging, it is important to acknowledge several limitations. Firstly, while the hand force production task may replicate everyday activities, the ankle dorsiflexion task may not fully represent typical movements due to the low force levels employed. Specifically, the task was conducted at 15% of MVC to minimize head motion within the MRI environment. However, this lower force level does not fully account for the higher force outputs required in more intense or dynamic activities, which may limit the task’s relevance to real-world scenarios. Nevertheless, it is important to note that fMRI studies focused on lower limb activity are rare because they face inherent constraints. The MRI environment restricts the types of tasks participants can perform, often favoring isometric contractions for lower limb studies to reduce head motion. In this study, excessive and consistent head motion during the force blocks led to the exclusion of four young participants from the originally planned 20. This challenge was likely related to the typically higher MVC observed in young participants during foot tasks. These findings serve as proof of concept and underscore the need for future research to develop and refine experimental paradigms that address the constraints of lower limb movements and minimize head motion within the MRI environment. It is also important to note that the isometric paradigms were initially designed to investigate these dynamics in individuals with PD. While the simplicity of the design aimed to align with tasks commonly used in PD research, we recognize the necessity of incorporating a range of conditions and MVC levels in future studies to better characterize motor performance and associated brain activity. However, caution should be exercised regarding the use of higher force levels, as excessive forces may lead to increased head motion and discomfort during fMRI scans. Future investigations could examine slightly higher forces and/or sustained contractions, as these may more accurately represent the force demands of real-world activities that often become increasingly challenging with age, such as maintaining balance. Understanding the neural circuits engaged under these varied scenarios is crucial for elucidating how the brain adapts to different force requirements and movement conditions. This knowledge can inform the development of targeted interventions designed to enhance motor performance in aging populations, ultimately improving their quality of life.

In summary, this study examined age-related differences in fMRI activity during isometric force control for hand and ankle tasks. While both YA and OA achieved similar force levels, there were changes in the dynamics of force production in OA. Additionally, OA exhibited greater and more widespread activation in non-motor regions compared to YA during both upper and lower limb force control tasks. Collectively, these findings suggest that aging impacts neural control of movement. Future studies should build on these findings to determine whether the observed changes are due to age-related declines, pathological conditions, or compensatory mechanisms.

## Data Availability

No datasets were generated or analysed during the current study.
